# Biofilm formation and multispecies interactions of bacteria recovered from drinking water systems in broiler houses and piglet nursery units

**DOI:** 10.1128/spectrum.01084-26

**Published:** 2026-09-11

**Authors:** Ulric Van Rossum, Marc Heyndrickx, Niels Demaître, Faizan Ahmed Sadiq, Nico Boon, Geertrui Rasschaert, An Cools, Koen De Reu

**Affiliations:** 1 Technology and Food Unit, Flanders Research Institute for Agriculture, Fisheries and Food 74875 , Merelbeke-Melle, Belgium; 2 Faculty of Bioscience Engineering, Centre for Microbial Ecology and Technology, Ghent University 26656 , Ghent, Belgium; 3 Faculty of Veterinary Medicine, Ghent University 26656, Ghent, Belgium; 4 Evap Proefbedrijf Pluimveehouderij, Geel, Belgium; 5 Advanced Therapies Group, School of Dentistry, Cardiff University, Wales, Cardiff, United Kingdom; 6 Inagro, Rumbeke-Beitem, Belgium; Innovations Therapeutiques et Resistances, Toulouse, France

**Keywords:** biofilm, multispecies, livestock, drinking water systems

## Abstract

The presence of biofilms on the surfaces of drinking water systems in livestock housing can compromise the microbiological quality of drinking water. Understanding the biofilm microbiome and the biofilm-forming capacity of its dominant species is therefore essential, particularly for identifying species that coexist and enhance biofilm formation through interactions. This study investigated 248 bacterial isolates representing the dominant microbiota of biofilm samples collected from drinking water systems in broiler houses and pig nursery units. Biofilm formation was assessed in both single-species cultures and 341 multispecies combinations. Of the tested isolates, 80% formed biofilms in single culture; however, only 25% were classified as strong biofilm formers. In multispecies combinations with up to four species or isolates, antagonistic or competitive interactions were dominant, whereas biofilm mass enhancement was observed in only a limited number of combinations. These included opportunistic pathogens, with *Citrobacter freundii* and *Staphylococcus nepalensis* identified as key species driving the interactions. Our research highlights the importance of specific microbial interactions in biofilm development and provides a potentially practice relevant four-species model to guide future research aimed at improving strategies for controlling biofilms in livestock drinking water systems.

**IMPORTANCE:**

Ensuring the quality of drinking water on livestock production farms benefits animal production and health. Persistent biofilms on water line surfaces can lead to microbial contamination, resulting in lower-quality drinking water. This study examines bacteria commonly found in biofilms within livestock drinking water systems in pig nursery units and broiler houses. Most bacterial isolates can form biofilms in monoculture. Specific interactions among certain species primarily mediate bacterial interactions that enhance biofilm biomass in multispecies communities. Identifying these key interactions enables the development of a synthetic model to guide research on controlling biofilms in livestock drinking water systems.

## INTRODUCTION

Biofilms are sessile or non-attached aggregates of microbial communities encased in a self-produced or shared extracellular polymeric substance (EPS) matrix (1, 2). This matrix not only facilitates surface adhesion but also protects against environmental stressors, including cleaning procedures and disinfectants (3, 4). Microbial biofilms are present in drinking water systems (DWS), where they compromise water quality and pose health risks to humans and animals (5–8). Globally, uncontrolled biofilm formation results in economic losses across the medical and human health, home care, and the food industry, as well as in animal husbandry and DWS, with estimated annual losses of USD 4.3 billion and USD 90.4 billion, respectively (9). Current biofilm control strategies in the livestock industry rely on mechanical and/or chemical cleaning and disinfection products approved by the European Chemical Agency (10, 11). However, biofilms often persist after cleaning and disinfection, with opportunistic pathogens present among other microorganisms (10). Biofilms on DWS surfaces consist of complex, multispecies microbial communities that may include both pathogenic microorganisms, such as *Pseudomonas aeruginosa*, *Bacillus cereus*, and *Clostridium perfringens*, as well as non-pathogenic species (7, 8, 12). Species-species interactions have been described in various environments and occur through complex metabolic exchanges that can be neutral, synergistic, or positive, promoting growth and persistence, or antagonistic, through nutrient competition or antimicrobial production (13–15). Although these interactions have not yet been investigated in DWS within livestock production systems, they are likely to play a key role in shaping microbial biofilm communities and persistence in this context. Furthermore, keystone species within these microbial communities can strongly influence biofilm development and resistance to environmental stresses (16–18). A study investigating multispecies biofilms composed of isolates from marine algae demonstrated that synergistic interactions often confer enhanced tolerance to antimicrobial agents, frequently due to matrix effects and protection conferred by secreted factors from neighboring organisms (19).

The persistence of biofilms in DWS underscores the need to analyze community composition and interspecies interactions to inform targeted monitoring and control strategies. While most studies of multispecies biofilm interactions have focused on isolates recovered from soil, human fecal samples, and food or food-processing environments (20–22), comparatively little is known about interactions among species originating from DWS surfaces in primary animal production environments (23–25). Our previous study conducted in broiler houses and pig nursery units showed that, in both sectors, *Staphylococcus* and *Pseudomonas* were the most frequently isolated genera from biofilm samples collected from the internal surfaces of DWS. *Staphylococcus saprophyticus* was the most common species across both sectors. In broiler houses, *P. aeruginosa* and *Stenotrophomonas maltophilia* were the next most frequent species, whereas in pig nursery units, *Pseudomonas fluorescens* and *Psychrobacter pulmonis* were the most prevalent (8). This study aimed to characterize the biofilm-forming capacity of isolates recovered from DWS biofilm samples in our previous study and to identify bacterial species that coexist and persist on DWS surfaces through species interactions within multispecies biofilms. These selected isolates will represent simplified synthetic model communities composed of a limited number of isolates, enabling the development of standardized multispecies biofilm models for further controlled experimental evaluation.

## MATERIALS AND METHODS

### Isolates used in this study

A subset of 248 bacterial isolates was selected from a collection of 1,284 isolates derived from the dominant microbiota of biofilms sampled at the end of a production round, before C&D, from the surfaces of DWS pipelines in 15 broiler houses and 15 pig nursery units, all located in Flanders, Belgium. Sampling was conducted at farmhouses that used either ground, surface, rain, or tap water. The selection was based on the most frequently identified species in each farmhouse, as described in previous research (8). From the pig nursery units, 120 isolates were selected, and from the broiler houses, 128 isolates were selected, ensuring representation from each farmhouse. Isolates from the broiler houses belonged to 26 genera and 53 species, whereas those from the pig nursery units comprised 26 genera and 49 species. This means that for some species, more than one isolate was selected. If this were the case, the letter I followed by a number was used to refer to the individual isolates. Species identification was previously performed by matrix-assisted laser desorption ionization and/or 16S rRNA gene sequencing (8).

### Biofilm quantification using crystal violet staining

#### Single-species biofilm quantification

Biofilm formation was quantified in polystyrene 96-well microtiter plates (Corning Inc., Corning, NY, United States, Coster 3596) and adapted from a previously described study (21). Briefly, bacterial isolates were plated on brain heart infusion (BHI) agar (Oxoid, Hants, United Kingdom, CM1136) and incubated for 72 h at 25°C. Afterward, five colonies were picked and inoculated into 50% BHI broth (Oxoid, CM1135) and incubated overnight at 25°C, then appropriately diluted in 50% BHI to an OD 610 nm of 0.1. This solution was then used to inoculate a 96-well microtiter plate, with 160 µL per well, in eight replicates. The plates were sealed and incubated for 24 h at 25°C. The temperature of 25°C was chosen based on the average temperature of the drinking water in the drinking water lines of broiler houses and pig nursery units (8). After the incubation, the plates were rinsed by immersion in tap water and air-dried at 37°C for 30 min. Biofilms were then stained with 200 µL of 0.1% wt/vol crystal violet (CV; Merck KGaA, Darmstadt, Germany, 101408) for 15 min at room temperature (RT), rinsed three times with water, and air-dried for 30 min at 37°C. Finally, 200 µL of 33.3% vol/vol acetic acid (Biosolve Valkenswaard, The Netherlands, 010741) was added to each well to solubilize the bound crystal violet. After a final 15 min incubation period at RT, the absorbance (Abs) at 595 nm was measured in a Multiskan FC Microplate Photometer (Thermo Fisher Scientific Inc., Waltham, United States, 51119100). Isolates were classified as non-, weak-, moderate-, or strong biofilm formers based on their OD values. The cutoff OD (ODc) was defined as three times the standard deviation above the OD mean of the blank control (Abs 595 nm = 0.12). After one independent experiment with eight replicates, the biofilm-forming capability was determined as follows: OD ≤ ODc = non-biofilm former, ODc < OD ≤ 2 × ODc = weak biofilm former, 2 × ODc < OD ≤ 4 × ODc = moderate biofilm former, and 4 × ODc < OD = strong biofilm former (26). In addition to blank controls, *Escherichia coli* MB5967 was used as a weak biofilm control, while *Microbacterium lacticum* MB6468 served as the strong biofilm control, as previously described (21).

#### Multispecies biofilm quantification

To test multispecies biofilm interactions, a second subset of isolates was selected based on the most dominant and commonly identified bacterial species in DWS biofilms across both production sectors (8). This resulted in the selection of 29 species. Subsequently, all possible four-species combinations among these 29 species, including those effectively isolated from biofilms in the drinking water line of the same sector and farmhouse, were evaluated for their four-species biofilm-forming capacity. This yielded 209 four-species combinations from broiler houses and 132 from pig nursery units. Biofilm formation was quantified in 96-well microtiter plates as described above. For each four-species combination, bacterial strains were mixed in equal volumes (40 µL each) to a total of 160 µL. The biofilm biomass of each multispecies combination was compared with the summed biomass of the corresponding monoculture biofilms. Synergy or positive interactions were defined when the average absorbance of the four-species biofilm (Abs_595_ FS) was higher than the absorbance sum of the single-species biofilms (Abs_595_ SIS): Abs_595_ FS > Abs_595_ SIS or Abs FS/Abs SIS > 1 = synergy (21). Combinations with ratios between ≥0.9 and <1 after the first test were identified as potential synergistic interactions and subjected to two additional replicates. Also, combinations with a ratio >1 were repeated two more times. If all repetitions showed an average fold >1, the combination was marked as a synergistic or positive interaction. If the results were <1, the combination was considered antagonistic. Next, to better understand which bacterial species and strains play a key role in synergistic interactions, dual and three-community communities at a 1:1 ratio were studied for each of the four-species combinations that showed interactions. The last tests were repeated at three different time points, with each test done in eight replicates.

### Scanning electron microscopy

To further visualize the synergistic effect, the four multispecies biofilm combinations with the highest synergy were selected. Scanning electron microscopy (SEM) was performed at the Flemish Institute for Biotechnology (VIB) in Ghent, Belgium. The biofilms were developed on stainless steel (SS) coupons in 6-well plates (Corning Inc., 3516) by placing the coupons in each well containing 50% BHI, then incubating for 24 h at 25°C. Single strains from the combinations were also used to produce biofilms on the coupons. Thereafter, the biofilms formed on the coupons were studied using SEM. Coupon samples with developed biofilms were placed in a second 6-well plate and incubated in fixative (4% paraformaldehyde and 2% glutaraldehyde in 0.1 M sodium cacodylate buffer) for at least 1 h at room temperature (RT). The samples were washed three times for 5 min each by immersing them in 0.1 M sodium cacodylate buffer. The plates were placed on ice, and the buffer was replaced with 1% osmium tetroxide in 0.1 M sodium cacodylate buffer to enhance contrast and promote further fixation. After a 1 h incubation, the samples were washed three times with deionized water, then dehydrated through a graded ethanol series (30%, 50%, 70%, 95%, and twice in 100%) for 15 min each at RT. The samples were then processed in a critical point dryer (Leica EM CPD300). Finally, the samples were mounted on aluminum stubs using carbon adhesive tape, sputter-coated with a 5 nm layer of platinum (Quorum Q150 ES), and imaged using a Crossbeam 540 SEM (Zeiss) at 1.5 kV. The SEM images of single species biofilms from the same isolates were reused for comparison with multispecies biofilms, as they were analyzed under identical conditions.

### Statistical analysis

Statistical analyses of multispecies biofilm quantification were conducted in GraphPad Prism (v10.1) using one-way analysis of variance (ANOVA) with Tukey’s post hoc test to identify significant differences (*P* < 0.05). Fisher’s exact tests (2 × 2 contingency table) were used to evaluate associations between species presence or absence in synergistic versus non-synergistic combinations. *P* values were adjusted for multiple testing using the Benjamini–Krieger–Yekutieli false discovery rate (FDR) procedure with a threshold of *q* = 0.05.

## RESULTS

### Single-species biofilm formation of bacterial isolates

**Fig 1:**
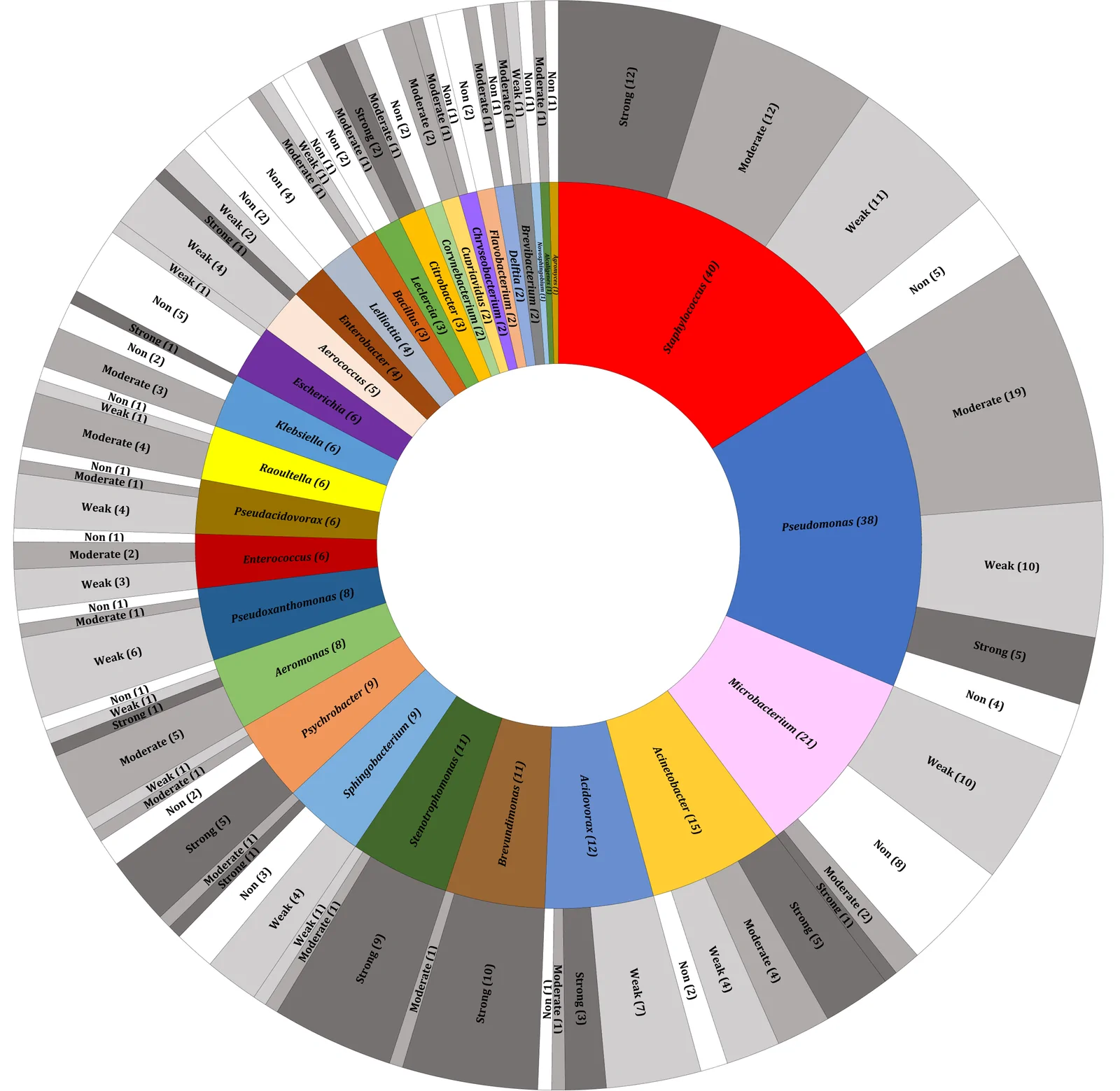
Classification of the biofilm-forming ability of the isolates at the genus level from pipeline contact surfaces of DWS from broiler houses and piglet nursery units at the end of a production round into non-biofilm formers, weak biofilm formers, moderate biofilm formers, and strong biofilm formers based on their OD values after crystal violet staining. The ODc was defined as three SDs above the mean OD of the blank control (OD595 = 0.12). Biofilm-forming ability was determined as follows: OD ≤ ODc = non-biofilm former, ODc < OD ≤ 2 × ODc = weak biofilm former, 2 × ODc < OD ≤ 4 × ODc = moderate biofilm former, and 4 × ODc < OD = strong biofilm former.

**Fig 2:**
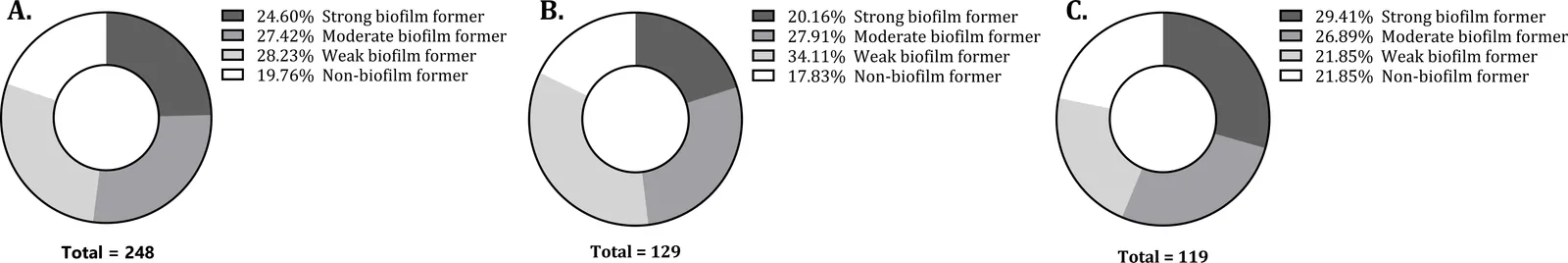
Classification of the biofilm-forming ability of the isolates (**A**) for both sectors, (**B**) for broiler houses, and (**C**) for piglet nursery units.

**Fig 3:**
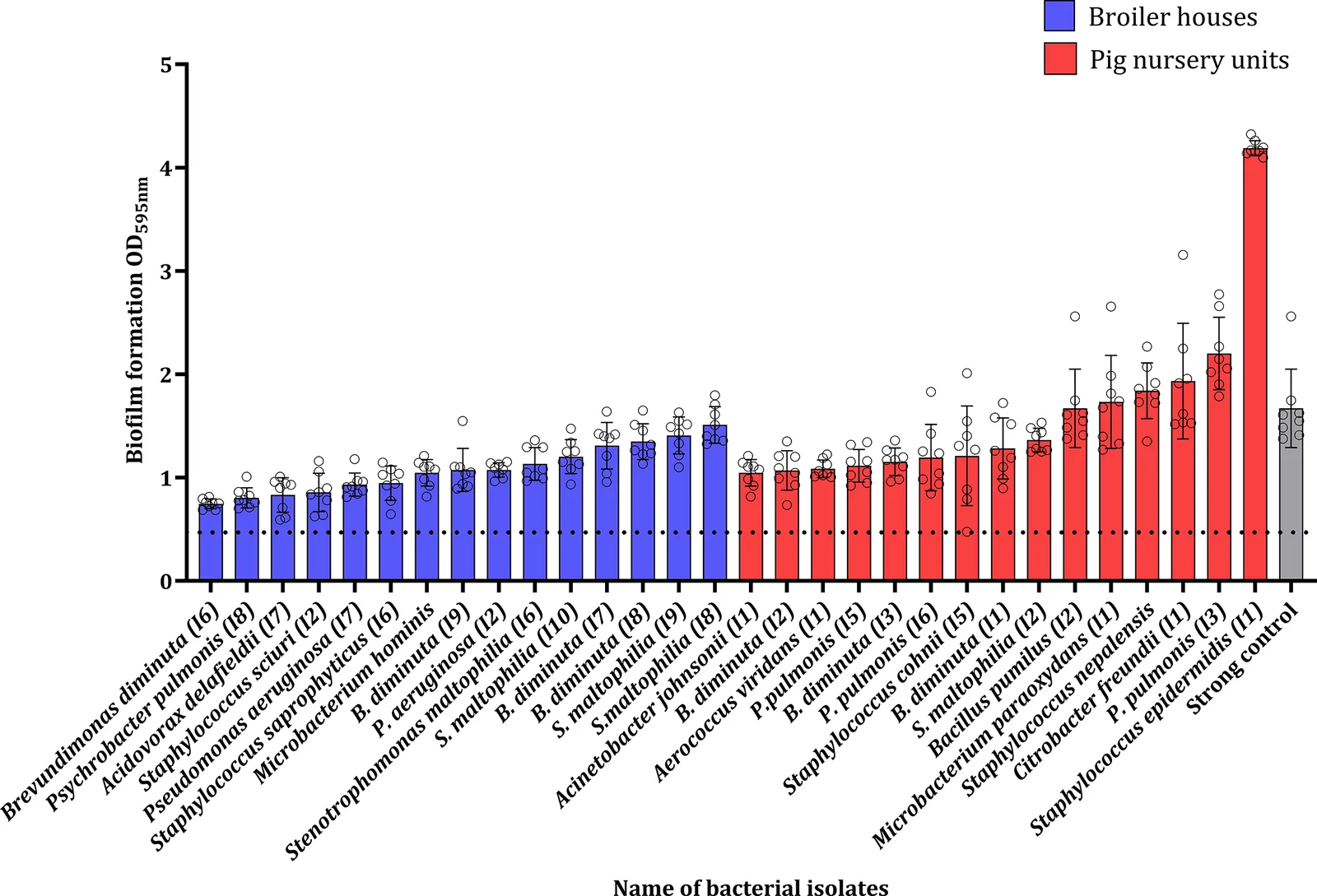
Top 30 isolates with the highest biofilm-forming capacity collected from DWS of broiler houses and piglet nursery units. The horizontal dotted line shows the cut-off OD value (OD_595_ = 0.47) for strong biofilm formation. The gray bar corresponds to the strong biofilm-forming control strain MB6468 of *Microbacterium lacticum.* When multiple isolates of a particular species were evaluated, the number in brackets indicates the isolate with the highest biofilm-forming capacity. Values represent the mean OD595 of *n* = 8 repetitions.

The classification of biofilm-forming capacity among the 248 bacterial isolates is shown in Fig. 1. Isolates were classified into four groups: non-biofilm formers, weak biofilm formers, moderate biofilm formers, and strong biofilm formers (File S2). Most isolates (~75%) were classified as non-, weak-, or moderate biofilm formers (Fig. 2A). Of the 248 isolates assessed, 61 were identified as strong biofilm formers (~25%), of which 26 (~20%; Fig. 2B) came from the broiler houses and 35 (~29%; Fig. 2C) from the piglet nursery units. In broiler houses, the predominant strong biofilm-forming species were *Stenotrophomonas maltophilia* (~31% of all isolates from broiler houses classified as strong biofilm formers) and *Brevundimonas diminuta* (~17%). In pig nurseries, *Staphylococcus* (~26%), including *Staphylococcus nepalensis*, *Staphylococcus saprophyticus*, and *Staphylococcus epidermidis*, was the predominant strong biofilm former. Other predominant strong biofilm formers included *Brevundimonas diminuta*, *Pseudomonas fluorescens*, and *Psychrobacter pulmonis*, each accounting for approximately 11% of the isolates in both sectors. Figure 3 shows the top 30 isolates with the highest biofilm-forming capacity collected from DWS of broiler houses and piglet nursery units. Across all isolates, a single *Staphylococcus epidermidis* isolate from pig nurseries was identified as the strongest biofilm former.

### Interactions of the multispecies biofilm of bacterial isolates

**Fig 4:**
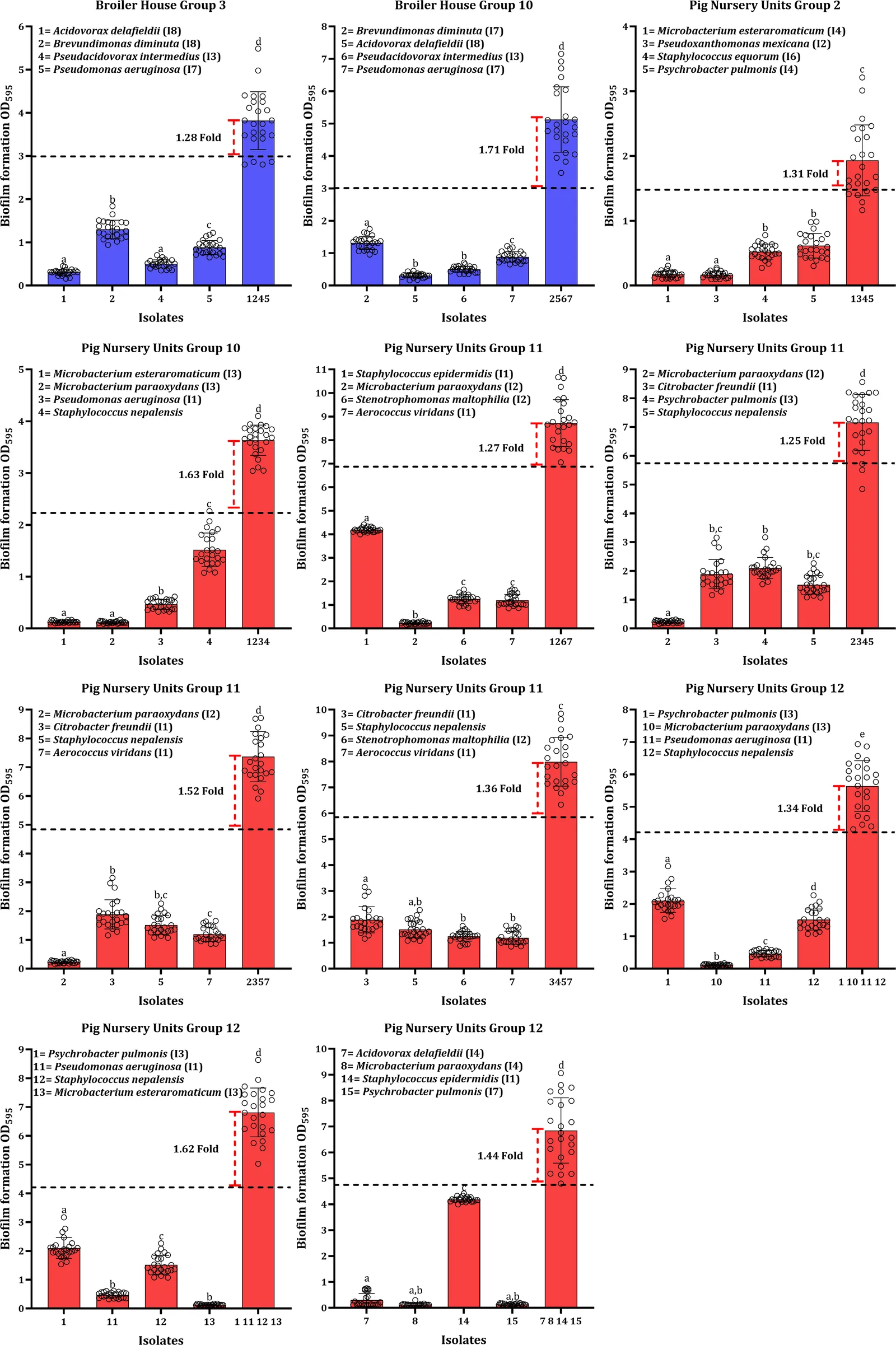
Interactions in four-species biofilm combinations with at least a 1.2-fold increase in biofilm mass compared with the sum of biofilm masses of all strains in isolation. These 11 groups belong to two groups of the broiler houses (blue) and the other nine from the piglet nursery units (red). In each four-species combination, comparisons were made among the biofilm masses of single species and the mixed-species biofilm using one-way ANOVA with Tukey’s test (*P* < 0.05 for significance). Values represent the mean OD595 of *n* = 24 repetitions after three independent experiments.

The 99 bacterial isolates from both sectors were grouped into 22: 10 for the broiler sector and 12 for the pig sector (File S1) four-species combinations, yielding 341 such combinations. These combinations were tested to identify those that exhibit synergy in biofilm mass. Based on the mean fold change across three independent experiments, only 15 of the four-species combinations (4%) showed a positive interaction or synergy, as indicated by significantly increased biomass relative to the sum of the biofilm masses of all single-species cultures (*P* < 0.05). Of these 15 combinations, 13 belonged to the pig sector. In contrast, only 2 combinations belonged to the broiler sector, and 11 showed a higher-than-average 1.2-fold increase in biofilm mass compared to the sum of the biofilm masses of all single-species cultures (Fig. 4).

### Specific bacteria involved in multispecies biofilm interactions

**Fig 5:**
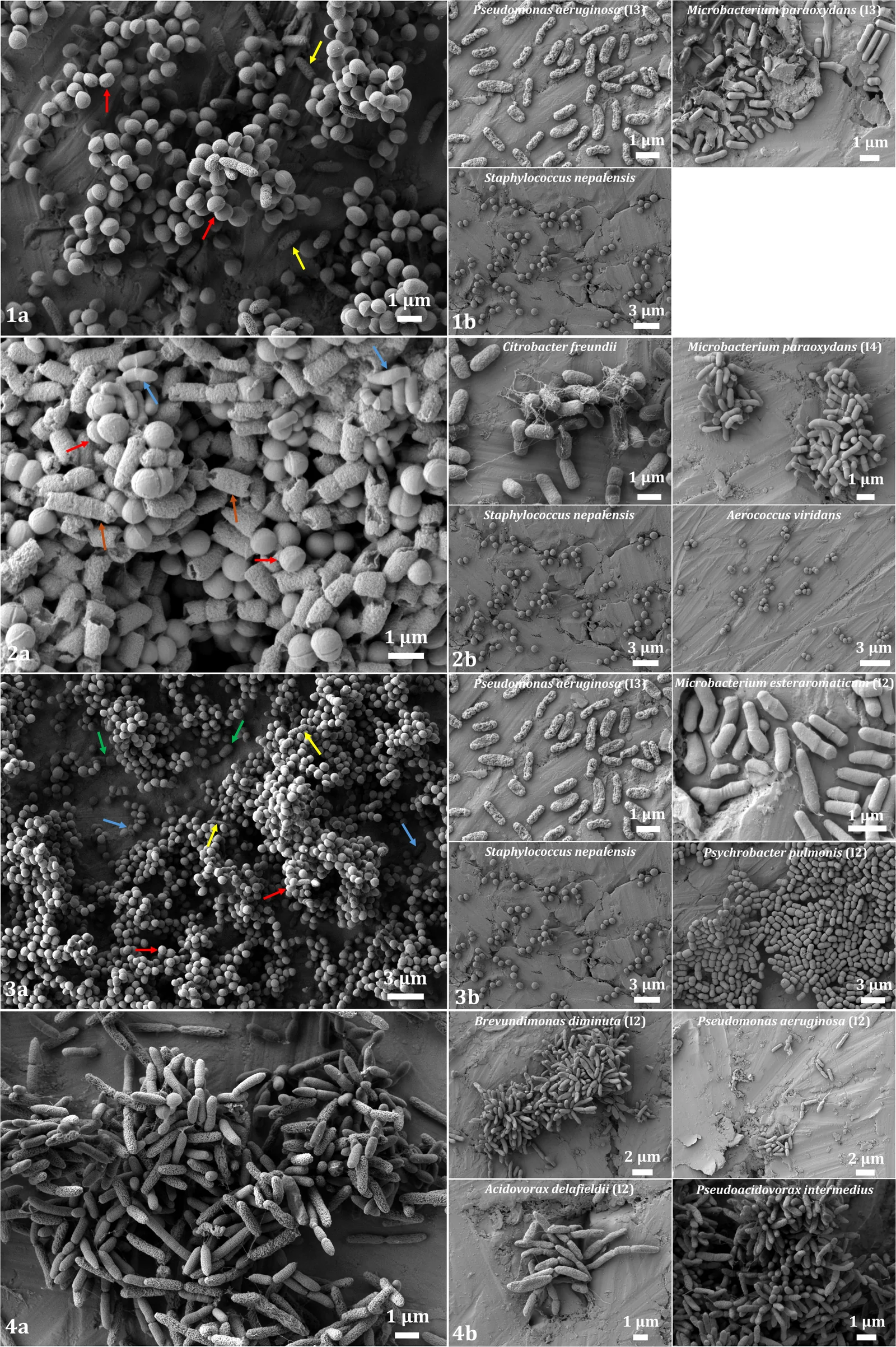
Scanning electron microscopy images of mono-species and multispecies biofilms, which showed interaction in biomass on stainless steel surface in 50% brain-heart-infusion medium after 24 h at 25°C. Panels **1a**,** 2a**, **3a,** and **4a **show the multispecies biofilm, while panels **1b**, **2b**, **3b,** and **4b** show the single-species biofilm. (**1a**) Combination of *P. aeruginosa* (I3), *S. nepalensis,* and *M. paraoxydans* (I3), where the species are shown with yellow and red arrows (*M. paraoxydan*s was not visible). (**2a**) Combination of *S. nepalensis*, *M. paraoxydans* (I3), *C. freundii*, and *Aerococcus viridans*, where the species are shown with red (both cocci), blue, and orange, respectively. (**3a**) Combination of *P. aeruginosa* (I3), *S. nepalensis, M. esteraromaticum* (I2), and *P. pulmonis* (I2), where the species are shown with yellow, red, blue, and green arrows, respectively. (**4a**) Biofilm combination between *B. diminuta* (I2), *P. aeruginosa* (I2), *Acidovorax delafieldii* (I2), and *Pseudacidovorax intermedius*. The same representative SEM images were utilized for the single species biofilms originating from the same isolates.

In the piglet sector, the strongest effect (average 1.63-fold increase in biofilm mass) measured by the crystal violet assay was observed in combination 1-2-3-4 from group 10, consisting of *Microbacterium esteraromaticum* (I2), *Microbacterium paraoxydans* (I3), *P*. *aeruginosa* (I3), and *S. nepalensis* (Fig. 4). Among these, only *S. nepalensis* was classified as a strong biofilm former in single-species cultures (Fig. 3), whereas the other three strains were not. SEM images of the three selected multispecies combinations from the pig nursery sector also showed higher biofilm coverage on the coupons than on single-species biofilms (Fig. 5(1) through 5(3)). In all three combinations, the same *S. nepalensis* isolate was included, and after 24 h it appeared to be the dominant species. In combination 1-2-3-4 from group 10, *M. paraoxydans* was not visually detected on the coupons, possibly due to loss during sample preparation or low abundance. In combination 2-3-5-7 from group 11, *Citrobacter freundii* and *S. nepalensis* appeared to be dominant. Finally, combination 1-11-12-13 from group 12 produced the highest biofilm coverage on the coupons (Fig. 5(4a)).

The highest level of interaction in the 96-well plates (average 1.71-fold increase in biofilm mass) among the four-species combinations was observed in combination 2-5-6-7 from group 10 of the broiler houses, containing *B. diminuta* (I2), *Acidovorax delafieldii* (I2), *Pseudacidovorax intermedius,* and *P. aeruginosa* (I2) (Fig. 4). Both *B. diminuta* and *P. aeruginosa* were classified as strong biofilm formers in single-species cultures. SEM images showed that individual species in the 2-5-6-7 combination had low coupon coverage in the biofilm. In contrast, their combination resulted in increased coverage across the entire coupon (Fig. 5(4)). Because all four species are rod-shaped, morphological differences among the individual species on the coupons could not be distinguished. The second interaction in the broiler houses involved the same species, but a different isolate of *B. diminuta* (I3) was used.

### Species shaping the multispecies interactions

**Fig 6:**
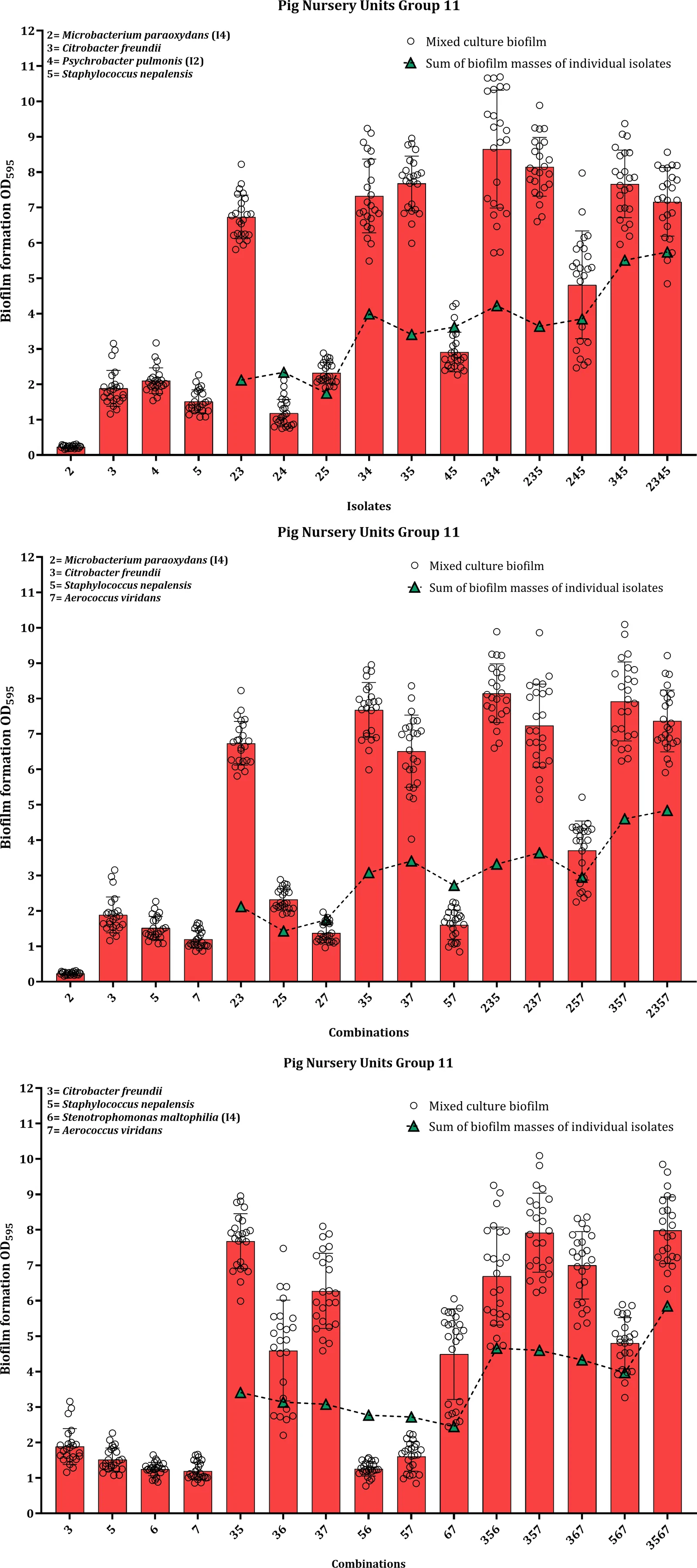
Three examples of all possible combinations of two and three species from a four-species combination with positive interactions containing the species *Citrobacter freundii*.

Among the 29 species included in the 341 four-species combinations, *C. freundii* (*P* = 0.0009, *q* = 0.0128) and *S. nepalensis* (*P* = 0.0001, *q* = 0.0028), each represented by a single isolate, were identified as key species driving interactions based on Fisher’s exact test with FDR correction (*q* < 0.05). *C. freundii* was present in 5 of the 15 combinations, and *S. nepalensis* was present in 8 combinations, only with pig sector isolates. These species interacted with *S. epidermidis*, *M. paraoxydans*, *P. pulmonis*, *Aerococcus viridans*, *S. maltophilia,* and *P. aeruginosa*. After evaluating all possible two- and three-species combinations from the 15 four-species combinations, *C. freundii* was the only key species in both cases (two-species: *P* = 0.0004, *q* = 0.0067; three-species: *P* = 0.0003, *q* = 0.0050), as illustrated in Fig. 6 and Fig. S1. These results further highlight the role of *C. freundii* in multispecies biofilm formation on DWS surfaces.

## DISCUSSION

### Single-culture biofilm formation of isolates collected from the DWS of broiler houses and pig nurseries

This study builds on our previous research on biofilm microbiota in DWS in broiler houses and pig nursery units. In that study, *Staphylococcus* and *Pseudomonas* were identified as the two dominant genera. *Staphylococcus* and *Pseudomonas* were the dominant genera, but species prevalence varied between sectors, indicating sector- and farm-dependent microbial ecology (8). Biofilm formation is influenced by both strain-level variability and environmental factors, such as water flow, nutrient availability, temperature, and chemical composition (1, 7, 21, 27–29). Assessing the biofilm-forming capacity of species from DWS offers insights into microbial ecology in these environments.

In the present study, 80% of the tested isolates formed biofilms in single culture, with 25% classified as strong biofilm formers. The proportion of strong biofilm formers was lower in broiler houses (20%) than in pig nurseries (29%). These differences between broiler houses and pig nurseries may reflect sector-specific factors, including water-use patterns and flow dynamics, water availability and chemistry, sanitation practices, as well as microbial differences between the two animal species (2, 8, 30–32). Another factor that may influence biofilm formation is the material composition of the piping system (33). In animal DWS, the quality of piping materials is generally subject to less stringent regulation than in human drinking water systems. However, further in-depth research is needed to better understand this difference. Among the tested isolates, recurrent species classified as strong biofilm formers were *S. maltophilia* in broiler houses; *S. nepalensis*, *S. epidermidis*, *P. pulmonis*, and *P. fluorescens* in pig nurseries; and *B. diminuta* and *S. saprophyticus* in both sectors. These species have commonly been found as dominant microbiota in biofilms in drinking water systems (8, 34). Most of these species are well-known biofilm formers that have been previously reported in the literature (10, 12, 35), except for *B. diminuta. B. diminuta* is a non-fermenting Gram-negative bacillus that has been isolated from water and human blood and is known to survive exposure to certain biocides (36, 37).

### Interactions in four-species biofilm combinations of isolates from broiler houses and pig nurseries drinking water line surfaces

Interactions with neighboring species strongly influence bacteria’s ability to establish and persist in biofilms. Microbial communities are shaped by interdependencies involving cell-to-cell aggregation, protection against biotic and abiotic stresses, and metabolic cooperation on surfaces (15, 38–41), thereby necessitating multispecies biofilm studies. Species-species interactions can be synergistic, antagonistic, or neutral: in synergistic interactions, one species promotes the growth of another. In contrast, antagonistic interactions occur when one species inhibits the growth or activity of another through competition for nutrients or by producing antimicrobial compounds (14, 42, 43). For example, a study using isolates from DWS in broiler houses reported that *Pseudomonas putida* inhibits *Salmonella* Java biofilm formation (23). Two other studies reported enhanced biofilm formation in dual-species interactions among isolates from DWS (24, 25). Despite nutrient availability, 96% of tested combinations produced weak, moderate, or no biofilm. This suggests that biofilm formation is not solely driven by nutrient availability but is often a response to environmental stress (biotic or abiotic) that increases EPS production, thereby enhancing survival (44, 45). Furthermore, biofilm formation can be highly dependent on specific species combinations. In natural systems, many microorganisms interact within relatively simple partnerships or small consortia, and the presence of additional species may disrupt these interactions, as some species may inhibit or exclude others within the community (16, 38, 44, 46, 47). Replicating natural microbial biofilm interactions in the laboratory is challenging due to multiple factors, including strain type, carbon source, nitrogen source, and mineral availability, that influence species interactions (7, 21, 46, 47). In addition, laboratory systems often lack the full biological context of natural environments, particularly the presence of uncultured or difficult-to-culture microorganisms that play important roles in shaping microbial interactions (7, 48). This may explain why only 4% of the four-species combinations exhibited cooperative or biofilm-synergistic interactions, corresponding to 1% of all tested combinations in broiler houses and 10% in pig nurseries (21, 49).

*C. freundii* and *S. nepalensis* were the two key species, each represented by a single isolate, in the four-species combinations. Furthermore, *C. freundii* was a key species in all dual- and three-species combinations within the 15 four-species interactions. *C. freundii* is a Gram-negative bacterium in the Enterobacteriaceae family, found in soil, sewage water, food, and the intestinal tracts of pigs, other animals, and humans, and is often associated with multidrug resistance (50–53). In our study, this isolate was classified as a strong biofilm former in monoculture. Previous studies have also reported that biofilm formation in *C. freundii* is associated with increased antimicrobial tolerance and multidrug resistance (54). Furthermore, *C. freundii* exhibited cooperative biofilm formation in multispecies communities, including interactions with enteroaggregative *Escherichia coli* and environmental species, such as *Delftia acidovorans,* isolated from drinking water (55, 56). Moreover, *S. saprophyticus* was the most abundant species in both sectors (8), and in our study, *S. nepalensis*, which is closely related to *S. saprophyticus*, was also identified as one of the main species involved in interactions. *S. nepalensis* is a coagulase-negative staphylococcus that has been isolated from the skin of various animals, including pigs, where it may function as an opportunistic pathogen (57, 58). The strongest interactions (>1.6-fold) occurred in four-species combinations that included the two key species and one of the dominant biofilm members (*M. paraoxydans*, *M. esteraromaticum*, *P. pulmonis*, *A. viridans*, or *P. aeruginosa*), whereas less abundant species likely played a more passive role (8). Although *M. paraoxydans* was not identified as a key species, its frequent involvement in interactions is consistent with previous reports highlighting *Microbacterium* spp. as keystone members of multispecies biofilms (16, 59, 60). The involvement of *P. aeruginosa* aligns with its established role as a biofilm-associated opportunistic pathogen (10, 61–63). Among the tested biofilm combinations for the broiler house isolates, the combinations consisted of *B. diminuta*, *A. delafieldii*, *P. intermedius*, and *P. aeruginosa*. These isolates are all Gram-negative bacteria belonging to Proteobacteria and are commonly found in soil and aquatic environments (37, 64–67). Overall, environmental biofilms are complex, involving physical and metabolic interactions that are difficult to reproduce in labs. Future research should focus on nutrient-poor solutions that reflect water conditions, temperature, gene expression, and metabolic molecules during biofilm formation. Biofilm biomass was only measured by crystal violet staining, which does not distinguish viable from dead cells (68). Additional research, such as cell counts, confocal laser microscopy, or molecular techniques, can offer deeper insights. To address biofilm issues in livestock DWS, key species along with their interacting partners can help develop biofilm models that mimic natural multispecies communities. In such communities, certain species function as major EPS producers and drive matrix formation, while others, although less abundant, depend on and contribute to the matrix environment and may play important roles in maintaining biofilm structure and function (69). Consequently, low-abundance or non-dominant species can have functional relevance that is not evident from community composition alone. This highlights the ecological relevance of multispecies biofilm models, which can be used to analyze disinfectants and other control strategies in *in vitro* biofilm systems (70).

### Conclusion

Biofilms in livestock DWS harbor diverse bacterial communities whose persistence is shaped by both intrinsic biofilm-forming capacity and interspecies interactions. Using isolates representing the dominant flora from broiler houses and pig nursery units, we show that most isolates formed biofilms in monoculture, although only 25% were strong biofilm formers. In mixed communities, antagonistic or neutral outcomes predominated, with synergistic enhancement of biofilm biomass observed in only a small subset of four-species combinations, more frequently in pig nursery-derived bacterial groups than in broiler-derived groups. Synergistic communities often contained opportunistic pathogens, and *C. freundii* and *S. nepalensis* were significantly represented among the combinations, with *C. freundii* also recurring in lower-order (two- and three-species) interactions. Together, these findings highlight that the development of strong multispecies biofilms on DWS surfaces is often constrained by competitive interactions under laboratory conditions, particularly in nutrient-rich media that do not fully reflect the nutrient-limited environments typically present in drinking water systems. Nevertheless, synergistic biofilm formation can emerge reproducibly in specific community contexts, suggesting that microbial interactions in natural environments may occur within defined, compatible species assemblages. Future work should evaluate these interactions under realistic DWS conditions (e.g., low-carbon water matrices, flow, temperature variations, and cleaning/disinfection regimes) using synergistic species combinations as synthetic model systems to develop targeted strategies to limit biofilm formation and persistence on DWS surfaces.

## Data Availability

The raw optical density measurements generated during this study are provided in the supplemental material.

## SUPPLEMENTAL MATERIAL

File S1 spectrum.01084-26-s0001.xlsx Biofilm formation data of multispecies.

File S2 spectrum.01084-26-s0002.xlsx Biofilm formation data of single culture.

Figure S1 spectrum.01084-26-s0003.docx All possible combinations of two and three species from a four-species combination with positive interactions.

ASM does not own the copyrights to Supplemental Material that may be linked to, or accessed through, an article. The authors have granted ASM a non-exclusive, world-wide license to publish the Supplemental Material files. Please contact the corresponding author directly for reuse.
