# Supplementary material for: Biofilm formation and multispecies interactions of bacteria recovered from drinking water systems in broiler houses and piglet nursery units
Source: Microbiol Spectr. 2026 Sep 11;14(10):e01084-26. doi: 10.1128/spectrum.01084-26 (PMC13689482; doi:10.1128/spectrum.01084-26)
Supplement: Supplementary file 1 [file spectrum.01084-26-s0003.docx]

Supplemental Material

**Fig A1** All possible combinations of two and three species from a four-species combination with positive interactions.
